# Sexism Interacts with Patient–Physician Gender Concordance in Influencing Patient Control Preferences: Findings from a Vignette Experimental Design

**DOI:** 10.1111/aphw.12193

**Published:** 2020-01-27

**Authors:** Dario Monzani, Laura Vergani, Silvia Francesca Maria Pizzoli, Giulia Marton, Ketti Mazzocco, Luca Bailo, Chiara Messori, Luca Pancani, Manuela Cattelan, Gabriella Pravettoni

**Affiliations:** ^1^ Applied Research Division for Cognitive and Psychological Science IEO European Institute of Oncology IRCCS Milan Italy; ^2^ Department of Oncology and Hemato‐oncology University of Milan Italy; ^3^ Department of Psychology University of Milan – Bicocca Milan Italy; ^4^ Department of Statistical Sciences University of Padova Padova Italy

**Keywords:** control preferences, gender differences, patient-centered care, sexism, shared decision making

## Abstract

**Background:**

Patient preferences regarding their involvement in shared treatments decisions is fundamental in clinical practice. Previous evidences demonstrated a large heterogeneity in these preferences. However, only few studies have analysed the influence of patients’ individual differences, contextual and situational qualities, and their complex interaction in explaining this variability.

**Methods:**

We assessed the role of the interaction of patient’s sociodemographic and psychological factors with a physician’s gender. Specifically, we focused on patient gender and attitudes toward male or female physicians. One hundred fifty‐three people participated in this randomised controlled study and were randomly assigned to one of two experimental conditions in which they were asked to imagine discussing their treatment with a male and a female doctor.

**Results:**

Analyses showed an interplay between attitude towards women and the gender of patients and doctors, explaining interindividual variability in patient preferences.

**Conclusions:**

In conclusion, patients’ attitudes toward the physicians’ gender constitutes a relevant characteristic that may influence the degree of control patients want to have and the overall patient‐physician relationship.

## Introduction

A good patient–physician relationship has a positive impact on clinical outcomes, while fostering information exchange and active participation of patients in medical decision‐making (Cooper‐Patrick, 1999; Coulter, 1997; Kaplan, Greenfield, & Ware, 1989; Little et al., 2001; Okunrintemi et al., 2017; Riedl & Schüßler, 2017). Several factors can influence this process. Among others, individual characteristics of both patients and physicians may interact to affect the quality of their relationship. The pivotal role of the patient–physician relationship has been stressed by the patient‐centered approach, a significant new perspective within the healthcare setting that has occurred in recent decades (Arnaboldi, Oliveri, & Pravettoni, 2015; Gorini, Mazzocco, & Pravettoni, 2013). This new paradigm points out the relevance of good communication between patients and healthcare providers as well as of the benefit of including patient preferences in the medical decision‐making process (Bailo, Guiddi, Vergani, Marton, & Pravettoni, 2019; Deber, 1994; Hashim, 2017; King & Hoppe, 2013; Strull, Lo, & Charles, 1984; Vick & Scott, 1998).

Shared decision‐making (SDM) has been identified as one of the core features of the patient‐centered approach (O’Connor et al., 2007; Elwyn et al., 2010; Patient Protection & Affordable Care Act, ). In SDM, healthcare providers and patients work together to make decisions about treatment by evaluating patients’ beliefs, needs, and preferences and considering evidence‐based clinical information about treatment options (Elwyn et al., 2010; Renzi, Riva, Masiero, & Pravettoni, 2016).

Essential core features of SDM are the elicitation and consideration of patient preferences for participation in decisions, the explanation of multiple choices and the evaluation of their pros and cons, the joint consensus on treatment‐related decisions, and mutual respect for these choices (Cutica, Vie, & Pravettoni, 2014). One of the primary steps of the SDM is the evaluation of patient preferences regarding the control and involvement they want during the decision‐making process. Control preferences do not express the extent to which patients wish to be informed; rather, they refer to the way patients want decisions to be taken. People may express variable degrees of decisional control in healthcare choices: while some patients want to actively participate, other people may not want to take responsibility for treatment‐related decisions (Arora & McHorney, 2000; Benbassat, Pilpel, & Tidhar, 1998; Degner, Sloan, & Venkatesh, 1997; Elwyn et al., 2012; Hanoch, Miron‐Shatz, Rolison, Omer, & Ozanne, 2015; Hubbard, Kidd, & Donaghy, 2008; Swenson, Buell, Zettler, & White, 2004). According to Degner and colleagues (1997), there are three main dimensions of control preferences: active, collaborative, and passive roles. Specifically, while those who prefer an active role want to make decisions on their treatment, patients preferring a passive role prefer their physician to make these decisions for them. Finally, patients opting for a collaborative role prefer to opt for a shared decision‐making approach with their doctor. A method to validly evaluate these differences is the Control Preference Scale (CPS) (Degner et al., 1997), an instrument consisting of five different vignettes, each reporting a different role that people may prefer to play in healthcare decision‐making.

The heterogeneity of patient preferences for control may be explained by considering internal and external factors. While the former include patients’ sociodemographic characteristics, dispositional factors, individual tendencies, and psychological resources, the latter include environmental, contextual, and contingent factors strictly related to the specific scenario of a given choice.

Several studies have investigated the influence of sociodemographic variables on patients’ control preferences and have demonstrated that factors such as age, gender, and education are among the most relevant predictors of the preference for being involved in medical decision‐making. For example, compared to men, women are more likely to prefer greater involvement in medical choices, playing a more active role or sharing treatment‐related decisions with their physicians (Arora & McHorney, 2000; Brom et al., 2014; Elkin, Kim, Casper, Kissane, & Schrag, 2007; Janz et al., 2004).

Only a few studies have considered the influence of people’s dispositional factors and psychological resources in shaping control preferences and confirmed the influence of adequate health literacy and numeracy on patient control preferences for a more active role in the decision‐making process (Hanoch et al., 2015; Moth et al., 2016; Seo, Goodman, Politi, Blanchard, & Kaphingst, 2015).

The influence of external factors on control preference heterogeneity has received relatively little attention as well. Thus, existing empirical evidence that confirms the role of illness severity in shaping patient control preferences is still weak: people seem to prefer a passive role or shared decision‐making rather than being actively involved in making medical choices when confronted with highly adverse clinical conditions (Benbassat et al., 1998; Degner, 1997; Efficace et al., 2014; Lechner et al., 2016; Stewart et al., 2000; Vick & Scott, 1998; Yennurajalingam et al., 2018).

Among external factors, the role of the physician’s gender is particularly relevant in shaping the patient–physician relationship, communication, and patient preferences (Cooper‐Patrick, 1999; Jahng, Martin, Golin, & DiMatteo, 2005; Robinson & Thomson, 2001; Roter, Hall, & Aoki, 2002). Compared to their male colleagues, women doctors are perceived as more empathic, more careful to build a positive relationship with patients, more likely to engage in positive talk, providing more information, and showing a stronger preference for collaborative decisions (Cooper‐Patrick, 1999; Garcia‐retamero, Wicki, Cokely, & Hanson, 2014; Kaplan et al., 1989; Krupat et al., 2000).

Building on this perspective, gender concordance, as an example of an interaction between internal (i.e. patient’s gender) and external (i.e. physician’s gender) factors, may play a significant role in shaping patient control preferences. In fact, gender concordance in patient–physician dyads has already proven to have a beneficial impact on a positive patient–physician communication, effective medical decision‐making, and patient satisfaction (Bertakis, Franks, & Azari, 2003; Garcia, Paterniti, Romano, & Kravitz, 2003; Gross et al., 2008). For example, a systematic review by Sandhu et al. (2009) demonstrated that same‐sex patient–physician dyads are characterised by higher levels of communication quality, namely, a special focus on the prevention and promotion of behavioral change and greater calm and submissiveness in paraverbal behavior, suggesting relative ease in the consultation. Thus, we may propose that gender concordance in patient–physician dyads could also influence patients’ control preferences by enhancing patients’ willingness to be more collaborative and involved in same‐sex dyads.

The presence of patients’ gender stereotypes and attitudes toward women may play a relevant role in predicting their preferred role in the clinical decision‐making process. Sandhu et al. (2009) claimed that non‐concordant gender dyads may be characterised by perceived differences in power, status, dominance, gender stereotypes, and attitudes toward the other sex that may lead to higher levels of tension and a lower communication quality. It is possible to hypothesise that sexism may partially explain the heterogeneity in patients’ control preference when consulting with a woman or a man physician. The ambivalent sexism theory (Glick & Fiske, 1996; Glick & Fiske, 2011) suggested that attitude toward women is a multidimensional construct that is not exclusively negative. Specifically, hostile sexism reflects an explicit antipathy toward women who do not conform to traditional gender‐stereotyped roles (e.g. homemakers, mothers), whereas benevolent sexism expresses seemingly positive but protective paternalistic beliefs about them. Hostile and benevolent sexism are moderately correlated aspects of stereotypes and attitudes toward women: the same person may have high hostile and low benevolent sexism at the same time, or the other way around. They both come from traditional ideas about the roles of women and justify inequalities in gender relationships, power, and status. Women who conform to gender‐stereotyped roles and do not threaten the power of men are the main targets of benevolent sexism. On the other hand, women who do not conform to traditional and stereotyped‐gender roles and threaten men’s dominance are objects of hostile sexism. Thus, while some women might be subject to hostile treatment, others are more likely to be protected and treated with benevolence. Since doctors are generally perceived as having higher status, more dominance, and more power, women physicians may be perceived negatively by highly hostile people. From this point of view, as also advanced by Klöckner Cronauer and Schmid Mast (2014), patients’ hostile sexism rather than benevolent sexism would more heavily influence the perception and evaluation of the woman physician and subsequently the degree of shared decision‐making the patients want during a consultation. Specifically, we hypothesised that male patients with a higher level of hostile sexism and who are consulting a woman doctor would exhibit the highest level of preference for an active role and the lowest degree of preference for passive and collaborative roles. This is because they would prefer to decide on their own (i.e. active role) rather than letting their woman doctor decide (i.e. passive role) or make the decision together with her (i.e. collaborative role).

## The Current Study

Given the beneficial effects of the shared decision‐making approach and of the congruence between preferred and actual patients’ roles in medical treatment‐related decisions, it would be helpful to identify the main internal and external predictors of heterogeneity in patient control preferences. This Randomised Controlled Trial (RCT) with stratified randomisation focused on the role of internal—both sociodemographic (i.e. people’s gender) and psychological ones (i.e. ambivalent attitudes toward women)—and external factors (i.e. doctors’ gender), as well as their interaction in shaping people’s preferences for the role they want to play in the decision‐making about their treatment for a mild medical condition. Specifically, to ensure that experimental groups were balanced on gender, participants were assigned to one of two conditions, using a stratified randomisation procedure in which both women and men participants were randomly assigned to one of two experimental conditions. In the first condition, participants were asked to imagine discussing their treatment with a male doctor, while in the second condition, they were asked to imagine a consultation with a woman physician. Participants’ hostile sexism and benevolent sexism were assessed. This RCT with a stratified randomisation design permitted us to test our hypothesis, namely, whether men with higher levels of hostile sexism and who were consulting a woman doctor exhibited the highest level of preference for an active role and the lowest degree of preference for passive and collaborative roles. Moreover, we advanced that benevolent sexism and its interaction with patient gender and physician gender would be less relevant in shaping patient control preferences. However, we did not have any specific hypothesis regarding the magnitude and direction of this possible interaction.

## Methods

### Participants and Procedure

A convenience sample of 153 participants was recruited with a snowball method. Volunteers were solicited by a group of undergraduate students to participate and were encouraged to ask their acquaintances to participate as well. The sample comprised 61 (39.9%) men and 92 (60.1%) women adults from northern Italy. The mean age was 40.32 (*SD* = 14.37) years (range: 18–78 years). Regarding occupational status, 28.2 per cent of the participants were white‐collar workers, 18.3 per cent were university students, 15.0 per cent were freelance workers, 2.6 per cent were blue‐collar workers, 17.6 per cent were healthcare professionals, 4.6 per cent were retired, 2.0 per cent were homemakers, 2.0 per cent were unemployed, and 9.9 per cent had another occupational status (e.g. trainee, journalist). Considering educational levels, most participants (35.9%) possessed a high‐school diploma, and 44.4 per cent possessed a university degree. A total of 2.6 per cent of participants had a lower educational level, and 17.0 per cent had a PhD or a postgraduate educational degree. Finally, 45.1 per cent of the participants were single, 49.7 per cent were married or lived with their partner, and 5.2 per cent were divorced. Participants completed an online consent form and an assessment of sociodemographic variables, control preferences, and hostile and benevolent sexism on Qualtrics.

### Measures

After completing the sociodemographic form, women and men were randomly assigned to one of two experimental groups via the randomisation procedure within Qualtrics. Specifically, in each experimental condition, participants received a specific form of the Control Preference Scale (CPS; Degner, Sloan, & Venkatesh, 1997), developed ad hoc to manipulate the gender of the consulted physician. The Control Preference Scale (CPS) was originally developed to evaluate the degree of control individuals want in making a decision about their health. It consists of five different vignettes, each reporting a different role people may assume in healthcare decision‐making. Each role is illustrated by a statement and a cartoon: “I prefer to make the decision about which treatment I will receive” (active role); “I prefer to make the final decision about my treatment after seriously considering my doctor’s opinion” (active‐collaborative role); “I prefer that my doctor and I share responsibility for deciding which treatment is best for me” (collaborative role); “I prefer that my doctor makes the final decision about which treatment will be used, but seriously considers my opinion” (passive‐collaborative role); “I prefer to leave all decisions regarding treatment to my doctor” (passive role). With graphic editor software, we modified the vignettes of the image‐revised self‐administered CPS (Solari et al., 2013). In both experimental conditions, the gender of the patient in each vignette matched the participant’s gender, whereas the gender of the consulted physician varied between the two conditions. In the first experimental condition (i.e. consulting a male doctor; *N* = 77), participants were presented with vignettes showing a male doctor. Moreover, they received the following instruction to complete the CPS: “Please imagine that you are feeling sick and you go to your doctor, Mario (i.e. Italian male first name) Rossi, to discuss your health. Your male doctor1In the Italian language, there are two distinct terms to indicate a man doctor (i.e. *dottore*) or a woman doctor (i.e, *dottoressa*). The same is true for other professions (e.g. *attore/attrice = *actor/actress; *cameriere/cameriera* = waiter/waitress). In the instructions of the CPS, “*dottore*” has been used to refer to a man doctor and “*dottoressa*” has been used to indicate a woman doctor. tells you about the main treatment options. Now, we will show you pairs of vignettes illustrating the options you and your male doctor may choose with respect to your therapy. Please pay attention to each vignette and choose the one you prefer between each pair.”

In the second experimental condition (i.e. consulting a woman doctor; *N* = 76), the modified vignettes of the CPS showed a woman doctor. The instruction to complete the scale had also been modified accordingly: “Please imagine that you are feeling sick and you go to your doctor, Maria (i.e. Italian woman first name) Rossi, to discuss your health. Your woman doctor tells you about some treatment options. Now, we will show you pairs of vignettes illustrating the options you and your woman doctor may choose with respect to your therapy. Please pay attention to each vignette and choose the one you prefer between each pair.”

Following the guidelines (Degner et al., 1997), we adopted the paired‐comparison administration method, in which participants were presented with ten successive comparisons (e.g. active role vs. active‐collaborative role, active‐collaborative role vs. collaborative role, etc.) of subsets of two of the five vignettes. In each comparison, participants were asked to choose the vignette they preferred. The two experimental conditions were homogenous regarding the main sociodemographic variables, namely, gender (*Χ* ^2^(1) = 0.05, *p* = .817), age (*t*(150) = −1.19, *p* = .235), educational level (*Χ* ^2^(3) = 5.59, *p* = .133), and marital status (*Χ* ^2^(3) = 3.03, *p* = .388). Comparisons between the two experimental groups are reported in Supplemental Material 1.

Finally, participants within each experimental condition answered the short version of the Ambivalent Sexism Inventory (Glick & Fiske, 1996; Glick & Whitehead, 2010). This 12‐item scale measures ambivalence in beliefs about women through two subscales assessing hostile sexism and benevolent sexism. “Women seek to gain power by getting control over men” and “women should be cherished and protected by men” are examples of items measuring hostile and benevolent sexism, respectively. Participants completed the Italian version of the short Ambivalent Sexism Inventory (Rollero, Glick, & Tartaglia, 2014). They had to indicate their agreement with each statement on a 6‐point Likert scale, ranging from 0 (i.e. strongly disagree) to 5 (i.e. strongly agree). In this study, the internal consistency was good (Cronbach’s alphas: hostile sexism = .82; benevolent sexism = .83), and the Pearson’s correlation between hostile and benevolent sexism (*r* = 0.57) was in line with previous findings (*r* = 0.53) reported in the validation paper by Rollero et al. (2014). Specifically, this correlation suggests that hostile and benevolent sexism are correlated but distinct aspects of stereotypes and attitudes toward women: the same person may have high hostile sexism and contemporaneously low benevolent sexism, or the other way around. Moreover, in our sample, benevolent and hostile sexism were weakly correlated with age (respectively, *r* = 0.20 and *r* = 0.27); while hostile sexism did not differ between men (*M* = 2.51; *SD* = 1.14) and women (*M* = 2.39; *SD* = 0.87) [*t*(151) = 0.72, *p* = .474], men reported higher levels of benevolent sexism (*M* = 3.00; *SD* = 1.11) than women (*M* = 2.61; *SD* = 1.05) [*t*(151) = 2.21, *p* = .028].

The study was conducted in compliance with the Declaration of Helsinki ethical standards. Informed consent was obtained from all participating subjects.

## Data Analysis

To evaluate people’s preferences for the five options of control preferences presented in the ten paired comparisons, we performed a log‐linear Bradley‐Terry (LLBT) model analysis using the R package Prefmod (Hatzinger, 2017; Hatzinger & Dittrich, 2012). The LLBT model was specifically developed to analyse paired‐comparison data and estimate people’s relative worth for each of the five options on a preference scale that sums to one. A greater preference for a specific role in treatment‐related decision‐making was indicated by a higher worth (*π*) score and the associated estimated probability (EP) of being preferred. Specifically, the EP indicates the probability of preferring option x in a comparison between x and the reference category y. For example, the EP of choosing option “active role” versus option “passive role” is computed as *π*_Active_/*(π_Active_* + *π_Passive_)* (see Hatzinger & Dittrich, 2012; Cattelan, 2012; and Dittrich & Hatzinger, 2009, for a detailed description of the LLBT model). Specifically, the llbtPC.fit and the llbt‐worth functions allowed for the testing of whether differences in people’s preferences for each role in the treatment‐related decision‐making process could be influenced by people’s sexism toward women, people’s gender, doctor’s gender, and their interactions. Because these two functions allow for the inclusion of only categorical covariates, hostile sexism and benevolent sexism were median‐split to represent low and high levels of hostile (median = 2.50) and benevolent sexism (median = 2.67). As stated above, even if hostile and benevolent sexism are correlated, they are different aspects of the multidimensional construct of sexism that may have differential influences on people’s preferences for control. For ease of interpretation of the results, we performed two distinct analyses, one for hostile and one for benevolent sexism, to test their influence on control preferences. The first analysis addressed the main and interaction effects of hostile sexism, people’s gender, and doctor’s gender. The second one evaluated the influence of benevolent sexism, people’s gender, and doctor’s gender on people’s preference for control in treatment‐related decision‐making. For each effect, the 95 per cent confidence‐intervals from bootstrapped resamples were computed using the R function boot. Significant effects were indicated by a *p*‐value lower than .05 and a 95 per cent confidence‐interval from bootstrapped resamples not containing zero. All statistical analyses were performed with R version 3.4.3 (R Core Team, 2019). A post‐hoc power analysis was performed to test the power of our models to detect the interaction between sexism, physician’s gender and people’s gender. In the two fitted models, the power of the tests for significance of the parameters related to various interaction effects was around 0.60 (moderate power) and, thus, our model may be unable to detect some other significant results in our analysis.

## Results

### People’ Preferences Regarding their Role in Treatment‐Related Decision‐Making

The role that was most preferred by participants was the collaborative role (i.e. C; *π *= 0.53), followed by the active‐collaborative role (*π *= 0.23), passive‐collaborative role (*π *= 0.18), active role (*π *= 0.04), and passive role (*π *= 0.03). Since the passive role was the least preferred option, it was chosen as the reference category in all subsequent analyses.

### People’s Hostile Sexism and Doctors’ and People’s Gender Explain Patients’ Preferences

We performed a first analysis assessing the influence of main and interaction effects of people’s hostile sexism, people’s gender, and physicians’ gender in shaping people’s preferences for their role in treatment‐related decision‐making. This analysis permitted us to test our main hypothesis that men with higher levels of hostile sexism and who are consulting a woman doctor would exhibit the highest level of preference for the active role and the lowest degree of preference for the passive and collaborative roles. Because the four‐way interaction was not statistically significant, it was removed, and a new model was analysed. The results of the final model are reported in Supplemental Material 2. These results indicate multiple and complex interactions between people’s hostile sexism, physicians’ gender, and people’s gender in influencing preferences for the active role, while the effect of interaction between people’s hostile sexism and physicians’ gender on the preferences for the active‐collaborative role does not depend on people’s gender. By considering both *p*‐values and 95 per cent BCI, four higher‐order interaction effects were significant. Regarding the active role, the interaction effects between people’s hostile sexism and gender (*estimate* = 0.61, *SE* = 0.28, *p* = .033, *95% BCI* [0.05, 1.28]) and between people’s hostile sexism and doctor’s gender (*estimate* = 0.63, *SE* = 0.27, *p* = .027, *95% BCI* [0.05, 1.29]) were responsible for differences in people’s preferences for making decisions autonomously from their doctor. Moreover, the interaction between people’s gender and physicians’ gender interacts to predict differences in the preferences for the active role (*estimate* = 0.67, *SE* = 0.29, *p* = .020, *95% BCI* [0.11, 1.31]). Regarding the active‐collaborative role, the effect of the interaction between people’s hostile sexism and doctor’s gender was responsible for differences in people’s preferences for making decisions autonomously after seriously considering their doctor’s opinion (*estimate* = 0.71, *SE* = 0.31, *p* = .023, *95% BCI* [0.05, 1.36]).

**Figure 1:**
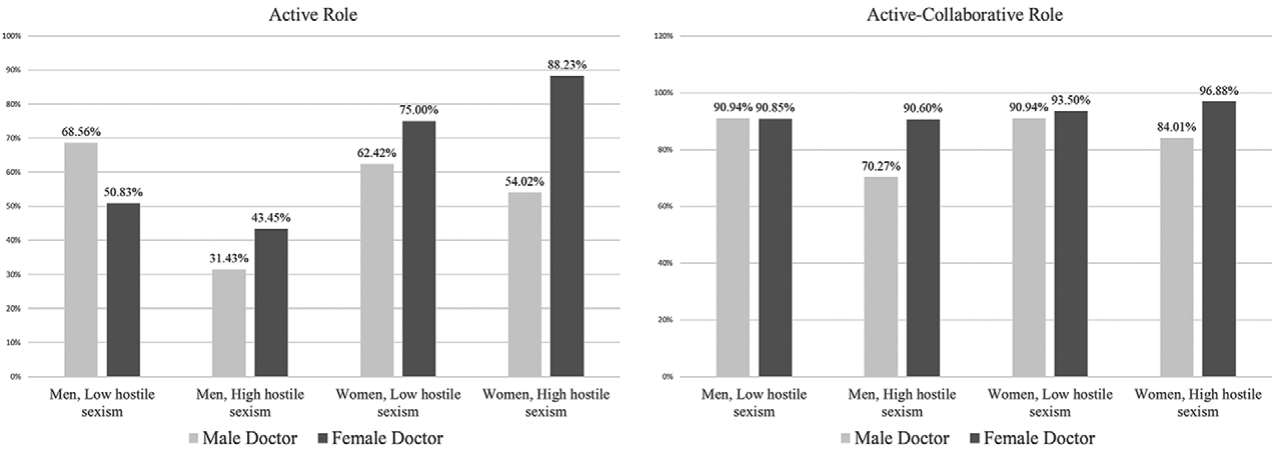
Preferences (estimated probability) for the active role versus the passive role (left side) and for the active‐collaborative role versus the passive role (right side) for women and men with low or high hostile sexism and who were consulting a man or woman physician.

Figure 1 reports differences in the levels of men’s and women’s preferences for the active role and the active‐collaborative role versus the passive role between women and men with low or high hostile sexism and in consulting a man or woman physician. As reported in the left side of the figure, women were more likely to prefer the active role versus the reference category “passive role” when consulting a woman physician (women with low hostile sexism: *π *= 0.05; EP = 75.0%; women with high hostile sexism: *π *= 0.05; EP = 88.23%) than did people collaborating with a male doctor (women with low hostile sexism: *π *= 0.04; EP = 62.42%; women with high hostile sexism: *π *= 0.04; EP = 54.02%). The pattern is more complex for men. Men with low hostile sexism had a higher probability of choosing the active role versus the reference category of passive role when consulting a male physician (*π *= 0.05; EP = 68.56%) than people working with a woman doctor (*π *= 0.02; EP = 50.8%). The opposite is true for men with high hostile sexism. Specifically, they were more likely to choose an active role when consulting a woman physician (*π *= 0.02; EP = 43.45%) than did people consulting a male doctor (*π *= 0.04; EP = 31.43%).

The results for the active‐collaborative role (right side of Figure 1) displayed a different pattern. Specifically, the preference for the active‐collaborative role versus the option “passive role” is stable for both men and women with low levels of hostile sexism consulting either a man (men: *π *= 0.21; EP = 90.94%; women: *π *= 0.23; EP = 90.85%) or a woman physician (men: *π *= 0.23; EP = 90.94%; women: *π *= 0.24; EP = 93.50%). In contrast, people with high levels of hostile sexism who were collaborating with a woman physician were more likely to prefer the active‐collaborative role versus the passive role (men: *π *= 0.26; EP = 90.60%; women: *π *= 0.23; EP = 96.88%) than did people consulting a male doctor (men: *π *= 0.20; EP = 70.27%; women: *π *= 0.18; EP = 84.01%).

### People’s Benevolent Sexism and Doctors’ and People’s Gender Explain Patients’ Preferences

The results of the analysis assessing the main and interaction effects of people’s benevolent sexism, people’s gender and physicians’ gender in shaping people’s preferences for their role in treatment‐related decision‐making are reported in Supplemental Material 2. This analysis permitted us to explore the interaction effects of people’s gender, doctor’s gender, and people’s benevolent sexism in shaping people’s control preferences. Two higher‐order interaction effects were significant. Specifically, the interaction was significant for the active role (*estimate* = −1.88, *SE* = 0.66, *p* = .004, *95% BCI* [−9.60, −0.62]) and the active‐collaborative role (*estimate* = −1.77, *SE* = 0.74, *p* = .017, *95% BCI* [−9.78, −0.11]). These results indicate that the interaction between people’s benevolent sexism and physicians’ gender that explains control preferences for the active role and the active‐collaborative role differs between men and women.

**Figure 2:**
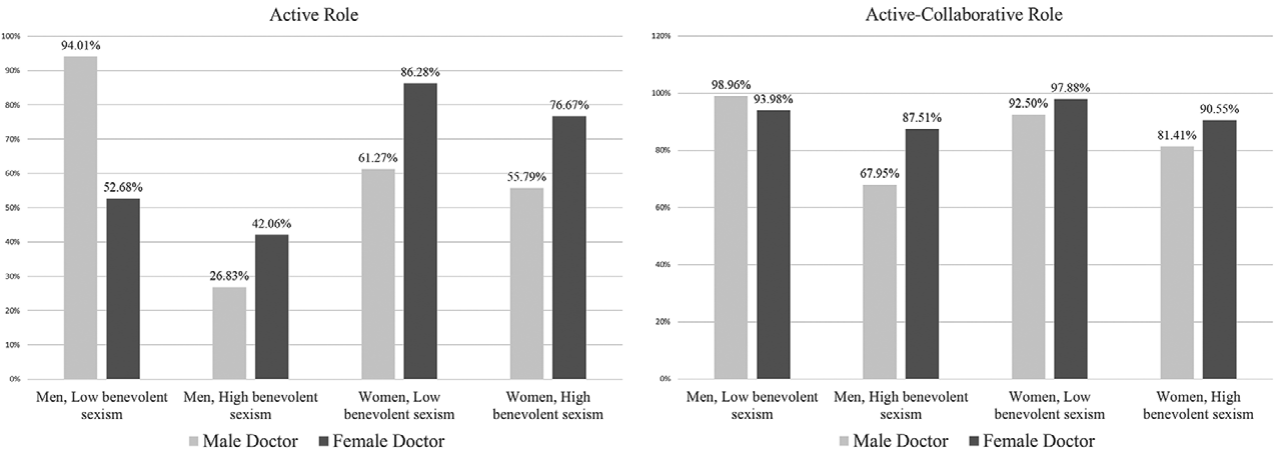
Preferences (estimated probability) for the active role versus the passive role (left side) and for the active‐collaborative role versus the passive role (right side) for women and men with low or high benevolent sexism and who were consulting a man or woman physician.

Figure 2 reports the differences in the levels of men’s and women’s preferences for each of the five roles in treatment‐related decision‐making between people with low or high hostile sexism and who were consulting a man or woman physician. As reported in the left side of the figure, men with low benevolent sexism and consulting a male doctor were more likely to prefer the active role versus the passive role (*π *= 0.04; EP = 94.01%) than men consulting with a woman physician (*π *= 0.02; EP = 52.68%). The opposite trend was found for men with high benevolent sexism. Specifically, they are more likely to choose an active role when consulting a woman physician (*π *= 0.02; EP = 42.06%) than people consulting with a male doctor (*π *= 0.03; EP = 26.83%). The pattern is simpler for women. Specifically, independent of levels of benevolent sexism, women tended to prefer the active role over the option “passive role” when consulting a woman doctor (low benevolent sexism women: *π *= 0.04; EP = 86.28%; high benevolent sexism women: *π *= 0.07; EP = 76.67%) than when consulting a male physician (low benevolent sexism women: *π *= 0.03; EP = 61.27%; high benevolent sexism women: *π *= 0.06; EP = 55.79%).

The results for the active‐collaborative role (right side of Figure 2) are quite similar. Specifically, independent of levels of benevolent sexism, women were more likely to prefer option B (active‐collaborative role) when consulting a woman doctor (low benevolent sexism women: *π *= 0.26; EP = 97.88%; high benevolent sexism women: *π *= 0.21; EP = 90.6%) than when consulting a male physician (low benevolent sexism women: *π *= 0.21; EP = 92.50%; high benevolent sexism women: *π *= 0.020; EP = 81.41%). We found a more complex pattern for men. Specifically, men with low benevolent sexism and consulting a male doctor were more likely to prefer option B over the reference category E (*π *= 0.23; EP = 99.96%) than men with low benevolent sexism consulting with a woman physician (*π *= 0.27; EP = 93.98%). The opposite is true for men with high benevolent sexism. Specifically, they were more likely to choose option B over option E when consulting with a woman physician (*π *= 0.23; EP = 87.51%) rather than a male doctor (*π *= 0.18; EP = 67.95%).

## Discussion

The elicitation and consideration of patient preferences for their role in medical decision‐making is one of the essential aspects of effective shared medical decision‐making (Cutica et al., 2014). The interprofessional relationship of the healthcare team is a fundamental part of promoting quality of care (Gilardi, Guglielmetti, & Pravettoni, 2014) and the patient–physician relationship and patient satisfaction are beneficially influenced when the doctor’s behaviors in the medical decision‐making process match the patient’s preferred or desired level of involvement and control (for a review, see Kiesler & Auerbach, 2006). Thus, the first essential step in the development of a good Shared Decision‐Making (SDM) model is to properly assess patient preferences for control and to better understand individual and contextual factors that may explain the tremendous heterogeneity in their desired role in treatment‐related decisions.

This study is the first attempt to shed light on the role of people’s sociodemographic and psychological variables and doctors’ gender in influencing people’s preferences for the role they want to play in the decision regarding treatment for a medical condition. Specifically, in an RCT with stratified randomisation we manipulated physicians’ gender with hypothetical scenarios to evaluate whether it may interact with people’s gender and people’s hostile and benevolent sexism to predict the desired role that people want to play in treatment‐related decisions for a mild clinical condition.

Our results demonstrated that people have a strong preference for a collaborative role in treatment‐related decision‐making. Specifically, the most commonly preferred role is the collaborative one, in which they collaborate and share decisions with their doctor when making a choice among possible treatments. Moreover, people reported strong preferences for options expressing a desire to decide independently after seriously considering the doctor’s opinions or to leave the decision to the doctors after their consideration of people’s opinions. Finally, people reported the lowest preferences for the most passive role, in which they leave all the decisions to their physician.

Participants showed a large heterogeneity in their preferences for control. In fact, subsequent analyses assessing the interplay between sexism, both hostile and benevolent sexism, and gender among both people and doctors showed that these interactive effects could explain interindividual variability in people’s preferences. Specifically, this empirical evidence showed that people with high levels of hostile sexism are more likely to play an active role and express a willingness to make decisions about their treatment by themselves or after considering their doctor’s opinion when consulting a woman physician. Thus, coherently with the ambivalent sexism theory (Glick & Fiske, 2001, 2001; Glick & Whitehead, 2010), we may advance that people with high hostile sexism would perceive a woman doctor negatively because she does not conform to the traditional stereotyped‐gender role and has high power, dominance, and status. It is likely that, because of high prejudice, highly hostile sexist people would have less trust in women physicians than less hostile people would and thus prefer a more active role and are not willing to delegate treatment‐related decisions to their doctor. Specifically, as hypothesised, men, in particular, holding hostile sexism prefer to decide on their own (i.e. active role). However, contrary to our main hypothesis, people with a high level of hostile sexism did not exhibit the lowest degree of preferences for the passive and collaborative modalities when consulting a woman doctor.

A secondary aim of our work was to explore the role of benevolent sexism in interacting with people–physician gender concordance in shaping people’s control preferences. Benevolent sexism had a relevant role in shaping people’s preferences for the control they want to have in deciding about their treatment. The results highlighted that people’s level of benevolent sexism interacted with the gender of both people and doctor in influencing people’s willingness to adopt an active role in the decision‐making process. In other words, the pattern of interaction between the doctors’ gender and people’s benevolent sexism was also dependent upon the patient’s gender. While women confronting a woman doctor were more likely to prefer to play an active role than were women consulting a male physician, the pattern for men was more complex and conditional upon benevolent sexism levels and physicians’ gender. Men with high benevolent sexism were more likely to prefer an active role when confronting a woman doctor, whereas men with low benevolent sexism showed a higher preference for deciding independently when consulting a male physician than did men consulting a woman physician. Thus, contrary to our hypothesis and to the belief of Klöckner Cronauer and Schmid Mast (2014), both hostile and benevolent sexism were relevant in influencing the perception and evaluation of a woman physician. Specifically, men who did not believe that only women conforming to traditional or gender‐stereotyped roles should be protected and treated with benevolence were less likely to prefer an active role when consulting a woman doctor than when consulting a male physician. These results may suggest that people with low benevolent sexism may have high trust and confidence in women doctors. Future research may better evaluate this pattern and shed more light on the process linking benevolent sexism, patient–doctor concordance, and patient preferences for being involved in making treatment‐related decisions.

Although this research clarifies the role of gender concordance and attitudes toward women in shaping patient control preferences, some limitations should be noted. First, because of the experimental nature of this study, people were asked to imagine consulting either a woman or a man physician and subsequently rating their preferred role in the treatment‐related decision‐making process. This approach can limit the ecological validity of our findings, and we may only suggest that these results may be observed in real‐life situations in which patients must discuss treatment options with their physician. Future studies may further investigate these aspects and evaluate whether and how patients’ gender and their hostile and benevolent sexism could influence control preferences when they consult their doctor, whether man or woman. Second, in our hypothetical scenario, we asked people to imagine themselves addressing an acute and mild medical condition. Further research should assess whether these results could also be generalised to hypothetical scenes and real‐life situations in which patients must cope with chronic or severe conditions. Third, Glick and Whitehead (2010) advanced that people may also hold prejudicial and ambivalent sexism toward men. Future studies should evaluate whether patients’ attitudes toward men alone and in interaction with doctor’s gender could shape their preferences for control in the medical decision‐making. Specifically, similar to hostile sexism toward women, we may advance that patients expressing hostile sexism toward men would prefer to decide on their own rather than letting their male doctor decide or make the decision together with him. Fourth, because of sample size issues and to facilitate the interpretation of results, we did not introduce other sociodemographic variables (e.g. age, educational level, etc.) into our model. Even though it was beyond the scope of our study to assess the role of other sociodemographic differences on control preferences, future studies should investigate whether these differences could moderate the identified influences. Fifth, because we recruited a convenience sample using a snowball method, these results may be not generalised to other samples and, thus, further inferences should be made only about this sample itself. Finally, as stated above, our models displayed only a moderate power. Thus, we might have been unable to detect some other significant results in our analyses. Further studies with enlarged sample sizes would be able to expand on the presence of significant effects among the variables considered in the present study.

Future research might evaluate the impact of patients’ other important internal and psychological factors on their control preferences. For example, it may be relevant to evaluate the role of individual differences that are strongly related to the elicitation of preferences or medical decision‐making, such as decision‐making style, rational and experiential thinking styles, risk propensity, and autonomy preferences (e.g. Deci & Ryan, 1987; Harren, 1979; Kasser & Ryan, 1999; Mellers & Cooke, 1996; Pacini & Epstein, 1999; Russo et al., 2019). Moreover, future studies may assess the impact of individual differences and psychological resources, such as dispositional optimism, self‐efficacy, coping styles, onset time delaying effect, and illness perception, which influence motivation, individual behavior, and decisions in both health contexts and everyday life situations (Arora, Weaver, Clayman, Oakley‐Girvan, & Potosky, 2009; Chawla & Arora, 2013; Greco et al., 2014; Monzani, Steca, Greco, D’Addario, Cappelletti, et al., 2015; Monzani, Steca, Greco, D’Addario, Pancani, et al., 2015; Pancani & Rusconi, 2018; Steca et al., 2015; Steca et al., 2017).

Despite these limitations, the current study reports several original findings that suggest important implications for further research and practical applications for the elicitation and evaluation of patient preferences for their involvement. Specifically, within the patient‐centered and SDM approaches (Arnaboldi et al., 2015; O’Connor et al., 2007; Elwyn et al., 2010; Gorini et al., 2013), the elicitation of the preferred role that patients want to play in treatment‐related decision‐making is the first necessary step of the SDM process. As also underlined by Kondylakis et al. (2014), this empirical evidence highlights the relevance of profiling patients’ sociodemographic and psychological factors to empower SDM. From this point of view, our results attested to the pivotal role of patients’ characteristics, both sociodemographic and psychological differences, and their interaction with physicians’ gender in explaining the heterogeneity of patient control preferences. Specifically, patient sexism may constitute a relevant characteristic that may influence the way patients interact with their physician, the quality of the interaction between doctors and patients, and patient satisfaction with their involvement in their treatment‐related decision‐making. In clinical practice, special attention should be paid to training physicians to effectively elicit patients’ preferred role in choosing among treatment options while keeping in mind the most relevant factors that contribute to the heterogeneity of these preferences. Women doctors should be aware that they may be negatively perceived and evaluated by highly hostile individuals, which subsequently may negatively impact the patient–physician relationship. As suggested by Klöckner Cronauer and Schmid Mast (2014), women doctors who address hostile patients may exert greater effort toward expressing an interest in patients’ beliefs, needs, preferences, and experiences with their diseases while involving and effectively engaging patients in the SDM process. Finally, specific psychoeducational interventions should be developed to target patients’ hostile attitudes toward women doctors, with the aim to emphasise to patients that gender discrimination can have a detrimental impact for outcomes of the care, healthcare availability, scientific progress and their overall experiences (Rotenstein & Jena, 2018). A secondary aim could be also to educate patients to have a better relationship, to adopt more effective communication modalities, to trust physicians and to be equally collaborative with both men and women doctors.

## Supporting information


**Material 1** **.** Descriptive statistics and results of comparisons between the two experimental conditions (i.e. man doctor and woman doctor)

**Material 2** **.** Results of the analysis assessing the main and interaction effects of hostile sexism, people’s gender, and physicians’ gender on preferences of control (significant effect in bold).

**Material 3** **.** Results of the analysis assessing main and interaction effects of benevolent sexism, participants’ gender, and physicians’ gender on preferences of control (significant effect in bold).
