# Supplementary material for: Sexism Interacts with Patient–Physician Gender Concordance in Influencing Patient Control Preferences: Findings from a Vignette Experimental Design
Source: Appl Psychol Health Well Being. 2020 Jan 27;12(2):471–92. doi: 10.1111/aphw.12193 (PMC7384069; doi:10.1111/aphw.12193)
Supplement: Supplementary file 1 — Material 1 . Descriptive statistics and results of comparisons between the two experimental conditions (i.e. man doctor and woman doctor) [file APHW-12-471-s001.docx]

Supplemental Material 1. Descriptive statistics and results of comparisons between the two experimental conditions (i.e., man doctor and woman doctor)

| Sociodemographic variable |  | Man doctor | | Woman doctor | | Statistical test |
| --- | --- | --- | --- | --- | --- | --- |
|  |  | Mean or % | sd | Mean or % | sd |  |
| Gender | Male | 38.97% |  | 40.78% |  | χ^2^(1) = 0.05, p = .817 |
|  | Female | 61.03% |  | 59.22% |  |  |
| Age |  | 38.93 | 13.25 | 41.71 | 15.37 | t(150) = - 1.19,  p = .235 |
| Educational level | Junior high school degree | 0.00% |  | 5.26% |  | χ^2^(3) = 5.59, p = .133 |
|  | High school degree | 32.46% |  | 39.47% |  |  |
|  | University degree | 48.05% |  | 40.80% |  |  |
|  | Postgraduate degree | 19.49% |  | 14.47% |  |  |
| Marital status | Single | 45.45% |  | 44.74% |  | χ^2^(3) = 3.03, p = .388 |
|  | In a stable relationship | 14.29% |  | 6.58% |  |  |
|  | Married | 36.36% |  | 42.11% |  |  |
|  | Divorced | 3.90% |  | 6.58% |  |  |
