# Supplementary material for: Sexism Interacts with Patient–Physician Gender Concordance in Influencing Patient Control Preferences: Findings from a Vignette Experimental Design
Source: Appl Psychol Health Well Being. 2020 Jan 27;12(2):471–92. doi: 10.1111/aphw.12193 (PMC7384069; doi:10.1111/aphw.12193)
Supplement: Supplementary file 2 — Material 2 . Results of the analysis assessing the main and interaction effects of hostile sexism, people’s gender, and physicians’ gender on preferences of control (significant effect in bold). [file APHW-12-471-s002.docx]

Supplemental Material 2. Results of the analysis assessing the main and interaction effects of hostile sexism, people’s gender, and physicians’ gender on preferences of control (significant effect in bold).

| Effect | Estimate (s.e.) | *p* | *95% BCI* |
| --- | --- | --- | --- |
| **Active** | **0.39 (0.18)** | **.029** | **[0.04, 0.77]** |
| **Active-collaborative** | **1.15 (0.20)** | **< .001** | **[0.78, 1.67]** |
| **Collaborative** | **1.70 (0.23)** | **< .001** | **[1.24, 2.33]** |
| **Passive-collaborative** | **0.68 (0.19)** | **< .001** | **[0.34, 1.10]** |
| Passive | 0.00 (NA) | NA | NA |
| **Active x hostile sexism** | **- 0.78 (0.25)** | **.002** | **[-1.35, -0.30]** |
| **Active-collaborative x hostile sexism** | **- 0.72 (0.26)** | **.006** | **[-1.37, -0.20]** |
| **Collaborative x hostile sexism** | **- 0.84 (0.29)** | **.004** | **[-1.56, -0.16]** |
| Passive-collaborative x hostile sexism | - 0.29 (0.25) | .243 | [-0.86, 0.24] |
| Passive x hostile sexism | 0.00 (NA) | NA | NA |
| Active x doctor’s gender | -0.37 (0.26) | .145 | [-0.94, 0.17] |
| Active-collaborative x doctor’s gender | - 0.01 (0.29) | .985 | [-0.63, 0.64] |
| Collaborative x doctor’s gender | - 0.13 (0.32) | .673 | [-0.84, 0.61] |
| Passive-collaborative x doctor’s gender | 0.42 (0.28) | .132 | [-0.14, 1.01] |
| Passive x doctor’s gender | 0.00 (NA) | NA | NA |
| Active x patient’s gender | -0.14 (0.22) | .544 | [-0.64, 0.32] |
| Active-collaborative x patient’s gender | 0.00 (0.25) | .999 | [-0.61, 0.52] |
| Collaborative x patient’s gender | -0.16 (0.28) | .563 | [-0.86, 0.44] |
| Passive-collaborative x patient’s gender | 0.43 (0.24) | .071 | [-0.06, 0.92] |
| Passive x patient’s gender | 0.00 (NA) | NA | NA |
| **Active x Hostile sexism x doctor’s gender** | **0.63 (0.29)** | **.027** | **[0.05, 1.29]** |
| **Active-collaborative x Hostile sexism x doctor’s gender** | **0.71 (0.31)** | **.023** | **[0.05, 1.36]** |
| Collaborative x Hostile sexism x doctor’s gender | 0.63 (0.34) | .065 | [-0.13, 1.38] |
| Passive-collaborative x Hostile sexism x doctor’s gender | 0.37 (0.31) | .230 | [-0.26, 1.04] |
| Passive x Hostile sexism x doctor’s gender | 0.00 (NA) | NA | NA |
| **Active x Hostile sexism x patient’s gender** | **0.61 (0.28)** | **.033** | **[0.05, 1.28]** |
| Active-collaborative x Hostile sexism x patient’s gender | 0.40 (0.31) | .192 | [-0.20, 1.16] |
| Collaborative x Hostile sexism x patient’s gender | 0.71 (0.38) | .036 | [-0.04, 1.56] |
| Passive-collaborative x Hostile sexism x patient’s gender | 0.04 (0.30) | .881 | [-0.61, 0.76] |
| Passive x Hostile sexism x patient’s gender | 0.00 (NA) | NA | NA |
| **Active x doctor’s x patient’s gender** | **0.67 (0.29)** | **.020** | **[0.11, 1.31]** |
| Active-collaborative x doctor’s x patient’s gender | 0.19 (0.32) | .556 | [-0.50, 0.89] |
| Collaborative x doctor’s x patient’s gender | 0.29 (0.35) | .388 | [-0.49, 1.07] |
| Passive-collaborative x doctor’s x patient’s gender | -0.28 (0.31) | .369 | [-0.96, 0.36] |
| Passive x doctor’s x patient’s gender | 0.00 (NA) | NA | NA |

*Note:* NA = estimate coefficients, standard errors, probabilities and bootstrapped confidence intervals are not estimated for the reference category “passive role”; 95% BCI = bootstrapped 95% confidence intervals.
