# Supplementary material for: Sexism Interacts with Patient–Physician Gender Concordance in Influencing Patient Control Preferences: Findings from a Vignette Experimental Design
Source: Appl Psychol Health Well Being. 2020 Jan 27;12(2):471–92. doi: 10.1111/aphw.12193 (PMC7384069; doi:10.1111/aphw.12193)
Supplement: Supplementary file 3 — Material 3 . Results of the analysis assessing main and interaction effects of benevolent sexism, participants’ gender, and physicians’ gender on preferences of control (significant effect in bold). [file APHW-12-471-s003.docx]

Supplemental Material 3. Results of the analysis assessing main and interaction effects of benevolent sexism, participants’ gender, and physicians’ gender on preferences of control (significant effect in bold).

| Effect | Estimate (s.e.) | *p* | *95% BCI* |
| --- | --- | --- | --- |
| **Active** | **1.38 (0.40)** | **< .001** | **[0.71, 8.72]** |
| **Active-collaborative** | **2.28 (0.45)** | **< .001** | **[1.49, 9.94]** |
| **Collaborative** | **2.84 (0.49)** | **< .001** | **[2.06, 10.86]** |
| **Passive-collaborative** | **1.14 (0.38)** | **.003** | **[0.59, 8.27]** |
| Passive | 0 (NA) | NA | NA |
| **Active x benevolent sexism** | **- 1.88 (0.44)** | **< .001** | **[-9.25, -1.12]** |
| **Active-collaborative x benevolent sexism** | **- 1.90 (0.48)** | **< .001** | **[-9.67, -1.06]** |
| **Collaborative x benevolent sexism** | **- 1.97 (0.52)** | **< .001** | **[-9.89, -1.07]** |
| Passive-collaborative x benevolent sexism | - 0.71 (0.42) | .090 | [-7.90, -0.00] |
| Passive x benevolent sexism | 0 (NA) | NA | NA |
| **Active x doctor’s gender** | **- 1.32 (0.46)** | **.004** | **[-8.78, -0.42]** |
| Active-collaborative x doctor’s gender | - 0.90 (0.53) | .090 | [-8.70, 0.27] |
| Collaborative x doctor’s gender | - 1.15 (0.57) | .045 | [-9.25, 0.12] |
| Passive-collaborative x doctor’s gender | 0.11 (0.47) | .819 | [-7.14, 1.28] |
| Passive x doctor’s gender | 0 (NA) | NA | NA |
| **Active x patient’s gender** | **- 1.15 (0.43)** | **.007** | **[-8.48, -0.39]** |
| **Active-collaborative x patient’s gender** | **- 1.02 (0.48)** | **.036** | **[-8.72, -0.12]** |
| **Collaborative x patient’s gender** | **- 1.12 (0.53)** | **.035** | **[-9.06, -0.20]** |
| Passive-collaborative x patient’s gender | 0.10 (0.43) | .821 | [-7.08, 0.88] |
| Passive x patient’s gender | 0 (NA) | NA | NA |
| **Active x benevolent sexism x doctor’s gender** | **1.66 (0.54)** | **.002** | **[0.66, 9.26]** |
| **Active-collaborative x benevolent sexism x doctor’s gender** | **1.50 (0.60)** | **.012** | **[0.17, 9.41]** |
| **Collaborative x benevolent sexism x doctor’s gender** | **1.56 (0.65)** | **.016** | **[0.22, 9.67]** |
| Passive-collaborative x benevolent sexism x doctor’s gender | 0.53 (0.55) | .333 | [-0.63, 7.88] |
| Passive x benevolent sexism x doctor’s gender | 0 (NA) | NA | NA |
| **Active x benevolent sexism x patient’s gender** | **1.77 (0.50)** | **< .001** | **[0.79, 9.22]** |
| **Active-collaborative x benevolent sexism x patient’s gender** | **1.38 (0.55)** | **.011** | **[0.30, 9.20]** |
| **Collaborative x benevolent sexism x patient’s gender** | **1.47 (0.60)** | **.014** | **[0.32, 9.42]** |
| Passive-collaborative x benevolent sexism x patient’s gender | 0.21 (0.49) | .667 | [-0.77, 7.46] |
| Passive x benevolent sexism x patient’s gender | 0 (NA) | NA | NA |
| **Active x doctor’s x patient’s gender** | **2.01 (0.54)** | **< .001** | **[1.04, 9.61]** |
| **Active-collaborative x doctor’s x patient’s gender** | **1.56 (0.63)** | **.013** | **[0.15, 9.52]** |
| **Collaborative x doctor’s x patient’s gender** | **1.73 (0.68)** | **.010** | **[0.23, 10.04]** |
| Passive-collaborative x doctor’s x patient’s gender | 0.25 (0.58) | .670 | [-1.09, 7.63] |
| Passive x doctor’s x patient’s gender | 0 (NA) | NA | NA |
| **Active x benevolent sexism x doctor’s gender x patient’s gender** | **- 1.88 (0.66)** | **.004** | **[-9.60, -0.62]** |
| **Active-collaborative x benevolent sexism x doctor’s gender x patient’s gender** | **- 1.77 (0.74)** | **.017** | **[-9.78, -0.11]** |
| Collaborative x benevolent sexism x doctor’s gender x patient’s gender | - 1.78 (0.80) | .026 | [-10.35, 0.13] |
| Passive-collaborative x benevolent sexism x doctor’s gender x patient’s gender | - 0.57 (0.69) | .414 | [-8.06, 0.96] |
| Passive x benevolent sexism x doctor’s gender x patient’s gender | 0 (NA) | NA | NA |

*Note:* NA = estimate coefficients, standard errors, probabilities and bootstrapped confidence intervals are not estimated for the reference category “passive role”; 95% BCI = bootstrapped 95% confidence intervals.
